# Immune Checkpoint Inhibitor-Induced Diabetes Presenting As Diabetic Ketoacidosis: A Case Report

**DOI:** 10.7759/cureus.114106

**Published:** 2026-08-07

**Authors:** Ravi Yadav, Kaijanee Kugavarathan, Victoria P John, Kiran Lama, Yousri Waziri

**Affiliations:** 1 Diabetes and Endocrinology, Hampshire Hospitals NHS Foundation Trust, Basingstoke, GBR; 2 Internal Medicine, Hampshire Hospitals NHS Foundation Trust, Basingstoke, GBR

**Keywords:** ctla-4 inhibitors, diabetic ketoacidosis (dka), immune-checkpoint inhibitors, immune-related adverse effects, ipilimumab-related adverse events, melanoma and immunotherapy, nivolumab-induced insulin-dependent diabetes, pancreatic beta cells, pd-1 inhibitors, type 1 diabetes mellitus (t1dm)

## Abstract

A 51-year-old woman with BRAF V600E-positive metastatic melanoma developed diabetic ketoacidosis (DKA) three weeks after commencing combination immunotherapy with nivolumab and ipilimumab, despite a normal baseline endocrine and metabolic assessment. She presented with vomiting, dizziness, and profound weakness.

Blood gas analysis demonstrated severe DKA (pH 7.09, bicarbonate 7.7 mmol/L, glucose 32.5 mmol/L, ketones 5.6 mmol/L), and laboratory evaluation showed markedly reduced C-peptide with positive anti-glutamic acid decarboxylase (anti-GAD) antibodies, consistent with fulminant, insulin-deficient diabetes secondary to immune checkpoint inhibitor (ICI) therapy. She was treated according to standard DKA protocols, transitioned to long-term insulin therapy, and her immunotherapy was de-escalated to single-agent nivolumab because of concern for recurrent endocrine toxicity.

This case illustrates the rapid and unpredictable onset of ICI-induced insulin-deficient diabetes, which occurred despite normal baseline investigations in this patient, and highlights the need for vigilant glucose monitoring in patients receiving combination checkpoint inhibitor therapy.

## Introduction

Immune checkpoint inhibitors (ICIs) are a class of immunotherapy that enhances the body's immune response against cancer by removing inhibitory signals that normally limit T-cell activation. By targeting immune checkpoint proteins, including cytotoxic T-lymphocyte-associated protein 4 (CTLA-4) and programmed cell death protein 1 (PD-1), these agents promote immune-mediated destruction of malignant cells and have revolutionised the treatment of advanced cancers, resulting in substantial improvements in survival across multiple tumour types [1].

Despite these therapeutic advances, ICIs are associated with immune-related adverse events (irAEs), with endocrine toxicities among the most clinically significant [2]. ICI-induced diabetes mellitus (ICI-DM) is a rare complication, occurring in fewer than 1% of treated patients, but is frequently permanent due to rapid and irreversible destruction of pancreatic β-cells by autoimmune mechanisms triggered by immune checkpoint blockade [3]. The condition often develops abruptly, even in patients with previously normal blood glucose levels, and commonly presents as diabetic ketoacidosis (DKA), a life-threatening metabolic emergency characterised by hyperglycaemia, ketosis, and metabolic acidosis. The risk of endocrine irAEs appears to be greater with combination immunotherapy, such as nivolumab (PD-1 inhibitor) and ipilimumab (CTLA-4 inhibitor), than with monotherapy. Current guidance therefore recommends routine blood glucose monitoring during ICI treatment to facilitate early detection and intervention.

This report describes the clinical presentation, diagnostic evaluation, and management of a patient who developed DKA following combination treatment with nivolumab and ipilimumab for advanced malignant melanoma. This case highlights the importance of vigilant monitoring, prompt recognition, and multidisciplinary management of immune-related endocrine toxicities in patients receiving immunotherapy, thereby supporting improved patient outcomes in the evolving era of cancer immunotherapy.

## Case presentation

A 51-year-old woman presented to the Emergency Department with multiple episodes of vomiting, dizziness, and generalised weakness. She also reported increasing lethargy but had no documented history of polyuria, polydipsia, or weight loss prior to presentation.

She had recently been diagnosed with malignant melanoma of the abdominal wall with adrenal metastasis. Molecular analysis demonstrated a BRAF V600E (Val600Glu) mutation. She was referred to the oncology service and commenced on combination immunotherapy with nivolumab (PD-1 inhibitor) and ipilimumab (CTLA-4 inhibitor). Baseline investigations before commencing treatment were unremarkable, including thyroid-stimulating hormone (TSH 5.34 mIU/L), random blood glucose (5.1 mmol/L), HbA1c (32 mmol/mol), cortisol (474 nmol/L), vitamin B12 (304 ng/L), and folate (10.9 ng/mL). Her presenting symptoms developed three weeks after the first cycle of immunotherapy.

Following the initial treatment cycle, she developed fever and pleuritic chest pain requiring therapeutic pleural drainage. Pleural fluid analysis demonstrated an exudative effusion with no evidence of malignant cells. She was treated with antibiotics and discharged. Shortly afterwards, she developed a widespread maculopapular rash that responded to antihistamine therapy.

Her past medical history was significant for superficial spreading melanoma of the right ear diagnosed in 1993, without evidence of invasion, which had been successfully treated with surgical excision. She had no personal or family history of diabetes mellitus.

On examination, she was alert with a Glasgow Coma Scale score of 15. She was tachypnoeic (respiratory rate 24 breaths/min) and tachycardic (106 beats/min) but remained haemodynamically stable (blood pressure 111/77 mmHg) and afebrile (36.6°C). She appeared tired, lethargic, and clinically dehydrated with dry mucous membranes. Cardiovascular and respiratory examinations were otherwise unremarkable. Abdominal examination demonstrated a localised tender lump above the umbilicus, while the remainder of the abdomen was soft and non-tender.

Point-of-care venous blood gas analysis demonstrated severe metabolic acidosis consistent with DKA, with a pH of 7.090, bicarbonate of 7.7 mmol/L, blood glucose of 32.5 mmol/L, and blood ketones of 5.6 mmol/L. Although the HbA1c at presentation was only mildly elevated (42 mmol/mol), this was consistent with the acute onset of ICI-DM occurring only three weeks after initiation of immunotherapy and therefore would not be expected to significantly influence long-term glycaemic markers. Further laboratory investigations demonstrated a markedly reduced C-peptide concentration and strongly positive glutamic acid decarboxylase (anti-GAD) antibodies, confirming acute insulin deficiency. The laboratory findings are summarised in Table 1.

**Table 1:** Laboratory investigations at presentation Bolded values represent the principal diagnostic abnormalities. The combination of severe metabolic acidosis (low pH and bicarbonate), marked hyperglycaemia, and elevated blood ketones is diagnostic of diabetic ketoacidosis. Markedly reduced C-peptide together with strongly positive anti-GAD antibodies supports immune checkpoint inhibitor-induced insulin-deficient diabetes mellitus. eGFR: estimated glomerular filtration rate; CRP: C-reactive protein; TSH: thyroid-stimulating hormone; GAD: glutamic acid decarboxylase; IA-2: insulinoma-associated antigen-2

Test	Lab Value	Reference Value
pH	7.090	7.370-7.450
Bicarbonate (HCO_3_⁻), mmol/L	7.7	21.0-28.0
Lactate, mmol/L	3.19	0.00-1.80
Glucose, mmol/L	32.5	3.9-6.4
Blood ketones, mmol/L	5.6	<0.6
Haemoglobin, g/dL	12	13-18
White blood cell count (×10^9^/L)	11.9	3,600-11,200
Platelet count (×10^9^/L)	243	140,000-440,000
Serum creatinine, µmol/L	67	45-84
Serum urea, mmol/L	4.3	2.8-8.1
eGFR, mL/min/1.73 m^2^	>90	>60
Serum sodium (Na), mmol/L	131	136-144
Serum potassium (K), mmol/L	4	3.7-5.2
CRP, mg/L	6.4	<10
Alanine aminotransferase (ALT), U/L	26	10-55
Amylase, U/L	9	28-100
TSH, mIU/L	0.21	0.24-4.2
Free thyroxine (fT4), pmol/L	24	11-22
C-peptide, pmol/L	57.6	350-1800
HbA1C, mmol/mol	42	<48
Anti-GAD antibodies, U/mL	>2000	0-10.9
Anti-tyrosine phosphate-like IA2 antibody	Negative	Negative

The patient was managed according to local DKA guidelines with intravenous fluids, fixed-rate insulin infusion, electrolyte replacement, and close biochemical monitoring. Following resolution of DKA, she was reviewed by the diabetes team and diagnosed with ICI-induced type 1 diabetes mellitus (T1DM) requiring lifelong insulin therapy. She was commenced on a basal-bolus insulin regimen and remains under regular follow-up with the community diabetes specialist nursing team.

Following multidisciplinary discussion with the oncology team, combination immunotherapy with nivolumab and ipilimumab was discontinued. Given the favourable oncological response and lower risk of severe irAEs with monotherapy, treatment was continued with single-agent nivolumab while maintaining close endocrine and oncological follow-up.

## Discussion

ICIs have transformed the treatment of advanced malignancies by enhancing anti-tumour immune responses through inhibition of immune checkpoint pathways, principally PD-1 and CTLA-4. However, this enhanced immune activation may also lead to irAEs, including endocrine dysfunction. Endocrinopathies are among the most frequently reported irAEs, with an estimated incidence of 3-40%, although ICI-DM remains rare, occurring in fewer than 1% of treated patients [4-8]. Despite its rarity, ICI-DM is clinically important because of its abrupt onset, irreversible insulin deficiency, and frequent presentation as DKA.

The clinical presentation of our patient is characteristic of ICI-DM. She had normal baseline glycaemic assessment before commencing immunotherapy, no personal or family history of diabetes mellitus, and developed DKA only three weeks after receiving her first cycle of combination nivolumab and ipilimumab. At presentation, she demonstrated severe hyperglycaemia, metabolic acidosis, ketonaemia, markedly reduced C-peptide concentrations, and strongly positive anti-GAD antibodies, consistent with acute autoimmune destruction of pancreatic β-cells. Although her HbA1c was only modestly elevated, this is readily explained by the rapid onset of insulin deficiency following initiation of immunotherapy and is a recognised feature of ICI-DM. As HbA1c reflects glycaemic control over the preceding two to three months, significant elevation would not be expected following only three weeks of hyperglycaemia.

The precise pathophysiological mechanisms underlying ICI-DM remain incompletely understood. PD-1/PD-L1 blockade is thought to activate autoreactive CD8+ T lymphocytes that infiltrate pancreatic islets, leading to rapid and irreversible β-cell destruction and profound insulin deficiency [9,10]. Unlike classical T1DM, islet autoantibodies are inconsistently detected in patients with ICI-DM, suggesting that the condition represents a distinct immune-mediated entity with overlapping autoimmune features [7,11]. In the present case, the markedly elevated anti-GAD antibody titre supported an autoimmune mechanism, although baseline islet autoantibodies were not measured. Consequently, pre-existing autoimmune susceptibility cannot be completely excluded, although the normal baseline glucose and HbA1c, combined with the close temporal relationship to immunotherapy, strongly support ICI-induced diabetes as the precipitating diagnosis.

Although both nivolumab and ipilimumab contribute to immune activation, current evidence suggests that PD-1 inhibition is principally responsible for ICI-DM. To date, diabetes has rarely been reported with CTLA-4 inhibitor monotherapy, whereas combination PD-1/CTLA-4 therapy is associated with a higher incidence of irAEs than either agent alone [1,8,12]. This observation is consistent with our patient's presentation following combination therapy. Importantly, clinicians should recognise that ICI-DM frequently develops abruptly without preceding classic symptoms of diabetes and may present directly with DKA despite previously normal metabolic investigations.

Alternative causes of DKA were considered during the diagnostic evaluation. The patient had no previous diagnosis of diabetes mellitus, no family history of diabetes, and no evidence of pancreatic metastases or pancreatitis. Although she had experienced a pleural infection several weeks before presentation, this had clinically resolved following antibiotic therapy; inflammatory markers were not suggestive of active infection, and the profound reduction in C-peptide together with strongly positive anti-GAD antibodies supported acute autoimmune insulin deficiency rather than stress hyperglycaemia alone. While the preceding illness may have contributed to metabolic decompensation, it is unlikely to fully explain the clinical and biochemical findings observed.

Following resolution of DKA, the patient required lifelong insulin therapy, reflecting the irreversible nature of β-cell destruction in ICI-DM. Current oncology and endocrinology guidelines do not recommend routine discontinuation of ICIs following recovery from DKA. Instead, decisions regarding continuation of immunotherapy should be individualised, balancing oncological benefit against the risk of further immune-related toxicity [2,12]. After multidisciplinary discussion, combination therapy was discontinued, and treatment was de-escalated to single-agent nivolumab. This decision reflected the patient's favourable oncological response while aiming to reduce the risk of further severe irAEs, with ongoing joint follow-up by oncology and diabetes specialist teams.

This case highlights several important clinical lessons. First, ICI-DM can develop rapidly despite normal baseline glycaemic investigations and may present with life-threatening DKA as the initial manifestation. Second, relatively modest HbA1c levels should not provide false reassurance in patients who develop acute hyperglycaemia shortly after commencing immunotherapy. Finally, clinicians caring for patients receiving ICIs should maintain a high index of suspicion when non-specific symptoms such as nausea, vomiting, lethargy, or dehydration occur. Early blood glucose testing, ketone measurement, and prompt assessment for DKA are essential to facilitate timely diagnosis and treatment. As the use of ICIs continues to expand across multiple malignancies, increased awareness of this rare but potentially fatal complication is essential to optimise patient outcomes.

## Conclusions

This case highlights that ICI-DM is a rare but serious irAE that may present abruptly as life-threatening DKA, even in patients with previously normal glycaemic assessment. Clinicians should maintain a high index of suspicion when patients receiving ICIs develop non-specific symptoms such as nausea, vomiting, lethargy, or dehydration, as prompt assessment of blood glucose and ketone levels may facilitate earlier recognition and treatment.

The profound reduction in C-peptide and ongoing insulin requirement observed in this patient are consistent with irreversible pancreatic β-cell dysfunction following ICI-DM. Decisions regarding continuation of immunotherapy after diagnosis should be individualised through multidisciplinary discussion, balancing oncological benefit against the potential risk of further irAEs.
